# Clinically Recognised Alcohol Use Disorder and Risk of Dementia Subtypes in 1.3 Million Women: A Prospective Cohort Study

**DOI:** 10.64898/2026.09.23.26363779

**Published:** 2026-09-29

**Authors:** Nasri G Fatih, David Hippisley-Cox, Klaus P Ebmeier, Joel Gelernter, Gillian K. Reeves, Sarah Floud, Maria D. Christodoulou, Anya Topiwala

## Abstract

**Background:**

Alcohol use disorder (AUD) is associated with increased dementia risk, but its long-term relationship with dementia, variation by age and subtype, and how this risk compares with that associated with alcohol consumption remain poorly characterised. We investigated these questions in a large prospective cohort of UK women.

**Methods:**

We analysed Million Women Study participants recruited aged 50–64 years and followed for more than 20 years through linked health records. Women with hospital-recorded AUD were matched to up to five controls. Cox models estimated associations between time-varying AUD and incident dementia overall, by subtype, attained age, with exposure lags of up to 15 years and competing-risk analyses. Self-reported alcohol consumption was analysed separately.

**Findings:**

Among 1,288,359 women, 20,070 with AUD were matched to 100,339 controls. Dementia occurred in 3,774 women with AUD and 8,239 matched controls. AUD was associated with all-cause dementia (HR=3·73[95% CI 3·52–3·95]), with strongest associations for mixed (5·03[3·88–6·51]), unspecified (4·29[3·92–4·70]), and vascular dementia (3·96[3·44–4·56]). Associations were substantially stronger before age 70 years (10·68[9·02–12·63]) than at ages 70-79 (3·75[3·47–4·04]) or ≥80 years (2·34[2·11–2·60]). Risk remained elevated with a 15-year lag (2·16[1·81–2·58]) and after accounting for competing mortality. Associations with self-reported alcohol consumption were substantially weaker.

**Interpretation:**

Clinically recognised AUD identifies women at markedly increased and prolonged dementia risk, particularly at younger ages and for vascular and mixed dementia, not captured by drinking quantity alone. People with AUD should be considered a priority population for earlier dementia prevention and risk reduction.

**Funding:**

Wellcome Trust Career Development Award to AT (306069/Z/23/Z); NIH grants P50AA012870 and R01DA054869 (JG). The Million Women Study is supported by the UK Medical Research Council (grant number UKRI1464)

## Full Text Availability

The license terms selected by the author(s) for this preprint version do not permit archiving in PMC. The full text is available from the preprint server.

